# Enormous, rapidly growing breast mass

**DOI:** 10.1186/s12885-015-2024-0

**Published:** 2015-12-24

**Authors:** Vivek Verma, Sanjay Muttineni, Rajesh R. Kulkarni, Edibaldo Silva-Lopez, William W. West, Robert B. Thompson

**Affiliations:** Department of Radiation Oncology, University of Nebraska Medical Center, 987521 Nebraska Medical Center, Ground Floor, Clarkson Tower, Omaha, NE 68198 USA; Department of Internal Medicine, University of Nebraska Medical Center, Omaha, NE USA; Department of Surgery, University of Nebraska Medical Center, Omaha, NE USA; Department of Pathology, University of Nebraska Medical Center, Omaha, NE USA

**Keywords:** Breast tumor, Phyllodes tumor, Radiotherapy

## Abstract

**Background:**

Signs and symptoms of a rapidly enlarging breast mass are not only important for all clinicians to recognize and assess, but also are not uncommon occurrences. We describe a similar but unique case that developed into an enormous, 36 cm exophytic mass.

**Case presentation:**

A 51-year-old woman with history of psychiatric conditions presented for signs and symptoms of sepsis. It was determined that the source was an enormous 36 cm mass originating from the breast/chest wall. After stabilizing the patient with antibiotics, she underwent successful resection. Surgical margins were positive, and histopathology demonstrated bland spindle cells with stromal overgrowth. Together with clinical and histopathological information, the patient was diagnosed with a phyllodes tumor.

**Conclusion:**

Differential diagnosis of rapidly growing breast masses is discussed, which are not uncommon occurrences in clinical medicine. One etiology, phyllodes tumors, can grow into large, exophytic masses as described. Oncologic treatment is discussed, usually consisting of surgery with postoperative radiotherapy for high-risk features.

## Background

The differential diagnosis of a rapidly growing breast mass is very important for not only oncologists, but any health care provider, owing to the relative ubiquity of the symptoms and need for further workup and treatment. In this report, we describe the exceptional case of a woman who noticed a rapidly growing breast mass that became extremely large. We discuss the difficulties of diagnosis as well as differential diagnoses of which clinicians should be aware.

## Case presentation

**Fig. 1:**
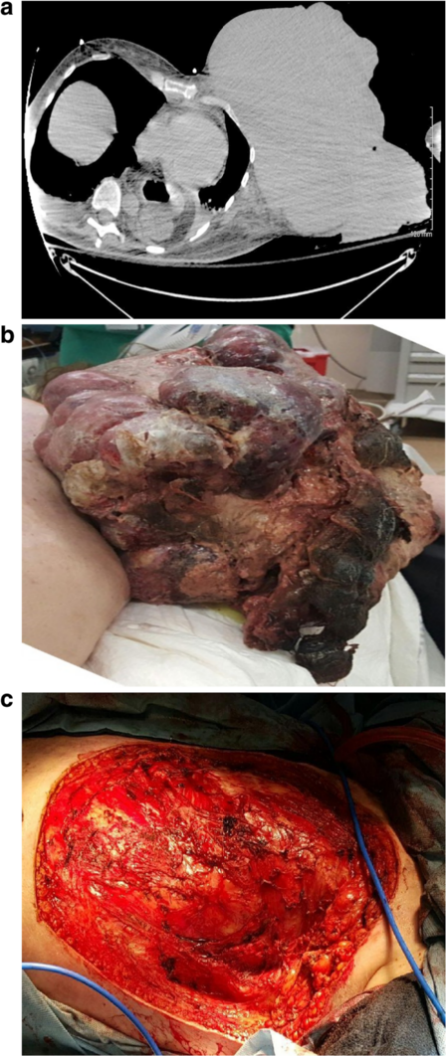
Gross appearance of the mass at presentation (**a**, *left panel*), computed tomography image without contrast of patient at presentation showing large exophytic mass (**b**, *center panel*), and postoperative appearance (**c**, *right panel*)

A 51-year-old woman with history of multiple psychiatric conditions including uncontrolled anxiety and depression presented to the intensive care unit with tachycardia and hypotension. She had initially felt a left breast mass five years ago but not sought medical attention, and the mass continued growing. Over the past sixteen weeks, the mass had nearly tripled in size and started oozing purulent fluid. Visual inspection revealed a 36 cm mass composed of several different colored, shaped, and textured tissues. Fluid drainage and necrotic debris was present (Fig. 1a) without axillary lymphadenopathy or other pertinent physical examination findings other than appearing ill with mental obtundation. She received fluid boluses and vasopressors. With further workup including elevated lactic acid level and white blood cell count of 31.6 thousand cells per microliter, broad-spectrum antibiotics were commenced. Computed tomography (CT) revealed a large, 36 cm exophytic mass in the left chest wall/breast with homogeneous density (Fig. 1b). Systemic staging using CT was negative.

After stability in clinical status was achieved, due to great concern of ongoing sepsis from the necrotic/infected tumor, emergent resection was done. Thus, pre-surgical biopsy could not be performed, although differential diagnoses included abscess/necrosis, sarcoma, phyllodes tumor, and fibroadenoma. Radical mastectomy was performed owing to intraoperative tumor involvement of the pectoralis and intercostal muscles. Owing to the emergent circumstances, large tumor size with necrosis, and no prior tissue diagnosis, nodal sampling was performed; lymph nodes were grossly nonenlarged. Owing to no clinical nodal disease as well as clinical suspicion for the aforementioned diagnoses, there was no indication for complete axillary dissection. Postoperatively a clean base was present (Fig. 1c) with skin grafted for wound closure aided by pressure dressings. The mass was sent for histological analysis.

**Fig. 2:**
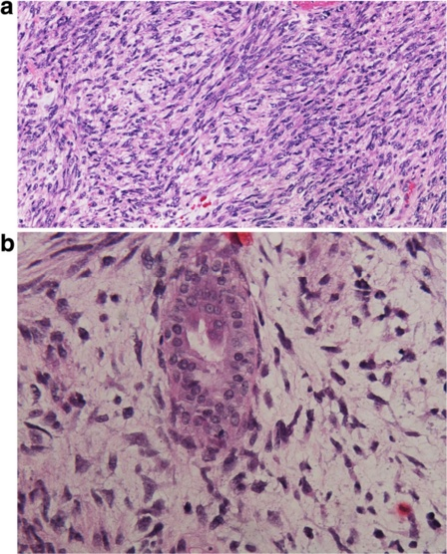
20x magnification of the resection specimen showing a cellular malignant spindle cell tumor with nuclear atypia but without evidence of differentiation (**a**, *left panel*), and 40x photomicrograph showing a rare benign gland in the midst of the malignant spindle cell tumor (**b**, *right panel*)

Pathological diagnosis of the mass was difficult. There was significant (over 50 %) necrosis with bland spindle cells being the only detectable cells (Fig. 2a). There was only one glandular/epithelial component seen (Fig. 2b) with significant stromal changes and high amounts of mitotic cells (45 mitoses per 10 high-powered fields). The deep margin of the tumor was positive as well. Pathological analysis demonstrated negative markers for neurofibroma, epithelial (including breast) carcinomas, melanoma, rhabdomyosarcoma, fibrosarcoma, or synovial sarcoma.

Whereas the pathological diagnosis was initially an unspecified spindle cell neoplasm, adding together the clinical history and presentation, the patient was diagnosed with a phyllodes tumor.

## Conclusions

Phyllodes tumors most often arise in patients in the 5^th^ decade of life and vastly more commonly in females [1]. They clinically present as a rapidly growing mass based in the breast, which is an important clue for diagnosis even if pathological diagnosis is inconclusive [2]. Spindle cellularity rules out fat necrosis and inflammatory breast carcinoma. Though only one epithelial (glandular) component was seen, the complete absence of such is less common in phyllodes tumors; likely, mesenchymal components of the tumor can overgrow the glanduloepithelial components, making the latter rare to find [1]. Phyllodes tumors are often confused with soft tissue sarcomas as well, including fibrosarcoma, which can complicate diagnosis. However, phyllodes tumors are vastly more common than primary breast sarcomas, occurring around 5–10 times more commonly, and 20–40 times more than primary breast fibrosarcomas [3]. Much of this involves placement of formerly-classified fibroadenomas into other categories, such as synovial sarcomas. Based on the WHO classification [4], this tumor was a malignant phyllodes tumor owing to high hypercellularity/pleomorphism, mitotic rate, invasive margins, and stromal overgrowth.

This patient was treated with postoperative radiotherapy, in part due to positive surgical margins, malignant phenotype, and stromal overgrowth. A large retrospective study of 478 patients with malignant phyllodes tumors demonstrated that tumors over 10 cm have recurrence rates of 15 % after mastectomy and worse survival, which was a primary consideration in proceeding with radiotherapy [5]. Moreover, if a positive surgical margin exists, it is known that stromal overgrowth in the tumor predicts for local recurrence (LR) [6] and distant failure (DF) [7], leading some to theorize that these patients may need systemic therapy as well, although this issue remains unresolved.

These are in contrast to the NCCN guidelines [8], which do not advocate radiotherapy or axillary staging, but the guidelines assume that a biopsy is performed to cement the diagnosis and do not account for emergent cases. The guidelines admit that the subtype of phyllodes tumor is less important than surgical margins, which has been corroborated by other studies to predict for poorer outcomes [6, 7]. Furthermore, large cohorts analyzed through the SEER database [9], which showed 15-year cancer-specific survivals (CSS) of 89 %, did not demonstrate radiotherapy to improve CSS, but due to lack of information could not assess radiotherapy’s effects on LR, especially with positive margins. On the other hand, a study from Europe [10] with 443 women demonstrated 17 % of patients with LR and 3.4 % with DF, most commonly to the lung. On multivariate analysis, factors associated with LR included residual disease/positive margins, borderline/malignant histology, and lack of radiotherapy. When specifically examining malignant/borderline tumors, radiotherapy use was associated with decreased LR. Factors associated with improved overall survival were tumors < 3 cm and absence of necrosis; for malignant tumors, use of mastectomy instead of breast-conservation was associated with improved overall survival. The latter finding conflicts with aforementioned data [9], but underscores the controversy in several aspects of treatment, especially in the rarer cases such as this patient.

This patient completed radiotherapy without wound healing complications but owing to psychiatric conditions and past issues of self-neglect, has failed to follow up.

## Consent

Written consent to participate and publish this case was obtained from the patient and is available on request. This case report was not required to be reviewed by the Institutional Review Board committee at the University of Nebraska Medical Center.

## Abbreviations

CSS: cancer-specific survival
CT: Computed tomography
DF: distant failure
LR: local recurrence
NCCN: National Comprehensive Cancer Network
SEER: Surveillance Epidemiology and End Results
WHO: World Health Organization
